# Dental Nurses

**Published:** 1917-12

**Authors:** Katherine H. Collings

**Affiliations:** Head nurse in the clinic of Northwestern University Dental School


### DENTAL NURSES

BY KATHERINE H. COLLINGS *

The system of serving the individual student, efficiently, in the various clinics, by means of young lady assistants, or nurses, was established at Northwestern University Dental School at the commencement of the 1916-17 term. The system has proved to be one of great value to the student body.

The mere presence of white-clad nurses, passing through the clinic, endeavoring to maintain aseptic conditions for the student to work under, places him in an environment which is a stimulus to careful work and promotes the formation of habits which make for a student's successful future.

The operative department of the general clinic, comprising about 150 chairs, is divided into three sections with a nurse in charge of each. Her station is at a cabinet which is fitted with a sterilizer and contains every material used by the student in this department. Her first duty in the morning is to place her particular section in readiness for the day's patients. Clean linen head rests are placed and fresh tray papers laid. Sterilizers are filled and kept at a boiling point constantly. The operative department cabinets contain gold, amalgam, cement, medicines, orthodontia supplies and an abundance of clean towels and tray papers. A student, upon the presentation of a cash order slip, is given the materials called for, in the proper amounts and a receipt for his patient. A filled tank, each, of grain and denatured alcohol, is at hand always for the use of the student. Broaches are dispensed on a slab containing phenol and alcohol, in short, everything that is for the use of student (and patient) is handled in a manner which

*Head nurse in the clinic of Northwestern University Dental School.

teaches him quantity values and efficiency in his operations. Antiseptic gauze, iodoform gauze, spirits of ammonia, and all other emergency materials are obtainable at each station.

The prosthetic department, containing about 100 chairs, is handled by one nurse, who dispenses every material used in the construction of crowns, bridges and dentures. A system whereby metal patterns are to be had, cut in every desirable size, eliminates lost time. Investment material is given out in correct quantities for investing a crown or inlay. Instruments, such as crown slitters, wedge cutters, repair outfits, etc., are at the student's disposal. Orders are given out by the nurse of this department, on the selected supply house, for teeth, the student selecting the size, shape, color, of his particular case. Just as in the operative department every need of the student working in this department is met.

The extraction and oral surgery clinic has the assistance of a nurse thoroughly familiar with forceps and all surgical instruments, their names and uses. She prepares the clinic and dates patients for the day. Her work is to mix all local anesthetic solutions, keep syringes filled, hand instruments to operator and student and to assist in the administration of gas. The advantage of an able assistant in a private office is very clearly shown here.

Oral surgery, peridental and operative clinics are all supplied with assistants who prepare for and work with the operator. This so enables the latter to save time that he is able to attend double the number of patients as heretofore.

Gradually, by the cooperation of faculty and nurse, changes are made, new ideas introduced and the system so becomes a perfect one which will always be remembered by the student as being an aid in many ways to his success. —*Northwestern Dental Journal.*

